# Non-invasive sensor methods used in monitoring newborn babies after birth, a clinical perspective

**DOI:** 10.1186/s40748-022-00144-y

**Published:** 2022-11-22

**Authors:** Oana Anton, Henry Dore, Elizabeth Rendon-Morales, Rodrigo Aviles-Espinosa, Paul Seddon, David Wertheim, Ramon Fernandez, Heike Rabe

**Affiliations:** 1 East Sussex NHS Healthcare Trust, Eastbourne, UK; 2 Brighton and Sussex Medical School, Academic Department of Paediatrics, Royal Alexandra Children’s Hospital, Brighton, UK; 3 Robotics and Mechatronics Systems Research Group, School of Engineering and Informatics, University of Sussex, Brighton, UK; 4 School of Computer Science and Mathematics, Faculty of Science, Engineering and Computing, Kingston University, Kingston upon Thames, UK; 5 Department of Neonatology, University Hospitals Sussex, Brighton, UK

**Keywords:** Heart rate monitoring, Newborn, Novel sensors

## Abstract

**Background:**

Reducing the global new-born mortality is a paramount challenge for humanity. There are approximately 786,323 live births in the UK each year according to the office for National Statistics; around 10% of these newborn infants require assistance during this transition after birth. Each year around, globally around 2.5 million newborns die within their first month. The main causes are complications due to prematurity and during delivery. To act in a timely manner and prevent further damage, health professionals should rely on accurate monitoring of the main vital signs heart rate and respiratory rate.

**Aims:**

To present a clinical perspective on innovative, non-invasive methods to monitor heart rate and respiratory rate in babies highlighting their advantages and limitations in comparison with well-established methods.

**Methods:**

Using the data collected in our recently published systematic review we highlight the barriers and facilitators for the novel sensor devices in obtaining reliable heart rate measurements. Details about difficulties related to the application of sensors and interfaces, time to display, and user feedback are explored. We also provide a unique overview of using a non-invasive respiratory rate monitoring method by extracting RR from the pulse oximetry trace of newborn babies.

**Results:**

Novel sensors to monitor heart rate offer the advantages of minimally obtrusive technologies but have limitations due to movement artefact, bad sensor coupling, intermittent measurement, and poor-quality recordings compared to gold standard well established methods. Respiratory rate can be derived accurately from pleth recordings in infants.

**Conclusion:**

Some limitations have been identified in current methods to monitor heart rate and respiratory rate in newborn babies. Novel minimally invasive sensors have advantages that may help clinical practice. Further research studies are needed to assess whether they are sufficiently accurate, practical, and reliable to be suitable for clinical use.

## Introduction

Reducing global new-born mortality is a paramount challenge for the humanity. There are approximately 786,323 live births in the UK each year according to the office for National Statistics; around 10% of these newborn infants require assistance in the period immediately after birth [1–4].

The main causes of death within the first after birth are prematurity and complications during delivery. Globally, 75% of neonatal deaths occur during the first week of life, with a substantial number dying within the first 24 h. For most newborn infants the transition from the intrauterine to the extrauterine environment occurs successfully; however, approximately 10% of newborn infants require assistance during this transition [4].

Heart rate (HR) is the most important clinical indicator to evaluate the status of a newborn and HR together with respiration form key components of the Apgar score, the predominant evaluative metric of newborn health and response to intervention. Therefore, to act in a timely manner and prevent avoidable damage, health professionals should be provided with accurate and timely monitoring of the main vital signs HR and respiratory rate (RR) [5, 6].

HR in those first minutes of life, especially in newborns needing resuscitation, may be used as a predictor of neonatal mortality and to determine the likelihood of developing moderate to severe brain injury in those who survive [4, 6]. HR at birth must be assessed quickly and accurately and is used as a guide to effective resuscitation and to evaluate other interventions such as placental transfusion [4, 5].

Frequently used methods to detect heart rate at birth include palpation and auscultation as previously recommended by international guidelines [6]. These are performed by trained medical professionals by feeling for pulsations at the base of the umbilical cord, feeling the brachial or femoral pulses and listening to the precordium with a stethoscope. Although auscultation seems to be superior to palpation both methods lack accuracy and only offer intermittent monitoring [7].

Other methods mentioned in the literature include pulse oximetry (PO), electrocardiography (ECG), Doppler ultrasound and forehead reflectance photoplethysmography (PPG) [7]. All these methods have their limitations and are affected by difficulties associated with the attachment of the sensing probes, movement artifacts and significant delay in display [7–9], also known as “time to HR”.

Respiratory rate (RR) is another key vital sign in neonatal monitoring [10]. Breathing rate or RR is defined as the number of movements that indicate inspiration and expiration of air per minute [11]. The normal respiratory rate of an infant is 30–60 breaths per minute [10, 11]. Respiratory distress is one of the most common reasons an infant is admitted to the neonatal intensive care unit. It is defined by the presence of one or more signs of increased work of breathing, such as increased respiratory rate (tachypnoea), nasal flaring, chest retractions, or grunting [12].

RR can be determined clinically by counting chest wall movement, but this is an intermittent measurement which is prone to inaccuracy and human error [11, 13, 14]. Continuous assessment of RR is possible by respiratory inductive plethysmography (RIP) or nasal airflow, but these methods require additional sensors and are not tolerated by acutely unwell babies [15]. There have been several publications looking at extracting RR from the pulse oximeter trace called plethysmogram (pleth) as a less invasive alternative [16–19]. Other authors investigated innovative sensors using Microwave Interferometric Radar Sensor or Impulse Radio Ultra-Wideband radar technology to monitor RR and detect apnoea in infants and preterm babies [20–25]. Although they have promising results and the advantages of non-contact monitoring, majority of these studies are in vitro experimental observational studies. To validate these results and assess their feasibility for clinical use they will need in vivo proof of concept studies and comparison with current gold standard.

Furthermore, researchers have sought opportunities to improve non-contact respiration monitoring such as radar, infrared imaging, and optical imaging [14]. These later technologies include thermal sensors and thermal imaging-based RR monitoring. These methods have not yet been approved for clinical environments [14].

Our previously published systematic review concentrated mainly on the clinical use of innovative neonatal HR monitoring sensors [8]. It identified the urgent need to develop non-invasive solutions to monitor both vital signs, HR, and RR, especially in vulnerable populations such as newborn babies [9, 19]. This current paper summarizes our previous findings and adds details regarding the clinical applications and basic technical details on piloted methods to monitor HR. It takes into consideration more recent publications and their potential impact on clinical practice. We also discuss a potential solution for continuous non-invasive RR monitoring in infants and young children using current established methods of monitoring [18, 19].

### Aims

The aim of this perspective is to provide basic technical information and clinical details of non-invasive novel sensors used to detect and monitor HR in the vulnerable newborn population. We highlight their advantages and limitations using the findings of our systematic review [8] and more recent publications that identify new technology [26–29]. A further aim is to discuss a potential solution for continuous non-invasive respiratory rate monitoring by extracting RR from the pulse oximetry traces of newborn babies [18, 19].

## Methods

We used data collected in our recently published systematic review to highlight the barriers and facilitators for the novel sensor devices in obtaining reliable HR measurements. Difficulties related to the application of sensors and interfaces, time to display, and user feedback were explored. The inclusion criteria for the systematic review were studies published in the last 15 years looking into the method to detect heart rate in both term and preterm infants in comparison to one of the established methods: PO or ECG. We investigated studies based on experiments, as well as feasibility studies in different health care settings: neonatal intensive care unit (NICU), high dependency unit (HDU), postnatal ward, delivery room and community birthing center. We also reviewed studies which have been registered on respective databases and are ongoing. Studies were excluded if there was no comparison with the gold standard in monitoring (ECG or PO) [8].

In addition, we reviewed the literature for more recent studies describing new dry ECG sensors used to detect and monitor HR in newborn babies [26–29].

Furthermore, we have summarized the findings of several feasibility studies using low pass filtering (LPF) to extract respiratory data from the raw pulse oximetry plethysmography traces of newborn babies, infants and young children [16, 18, 19].

## Results

The previously published systematic review looking into heart rate monitoring in newborn babies performed a full article analysis on twenty-six studies published in the previous 15 years. Most studies were observational and based on experiments. The population included term and preterm babies in Neonatal Intensive Care Units (15 studies). The other locations were the delivery room, neonatal high dependent units and postnatal wards [8].

Novel sensors, identified by the review, were designed to overcome the limitations of the established methods such as chest auscultation, cord palpation, ECG and PO. Auscultation and palpation, although rapid and easily available, have proven to be inaccurate which can affect the success of the resuscitation process [7, 8]. They can underestimate HR by a mean of 14 and 22 beats per minute respectively [30, 31].

ECG and PO are both more precise compared to clinical assessment alone but have their own disadvantages [10]. There are several publications that identify a significant delay in obtaining a reliable HR from birth which often exceeds one to two minutes [9, 30, 32, 33]. Other limitations are due to the potential damage to the fragile skin of preterm infants caused by adhesive electrodes which causes stress, pain and can lead to infection [34].

Novel technology development is aimed to minimize patient contact and produce non-obtrusive measurements of vital signs. The systematic review identified skin contact and non-contact sensors. The sensors using skin contact were reflectance photoplethysmography (rPPG), dry ECG electrodes, handheld doppler and digital stethoscopes. The non-contact sensors identified were camera photoplethysmography (PPG), capacitive sensors/electrodes [35], piezo-ceramic sensors piezo-electric sensors and laser Doppler vibrometer.

Please see Table 1 for a summary of these sensors.

**Table 1:** Summary of novel sensors to detect heart rate in newborn babies

Name (References)	Skin contact (SC) Non-Contact (NC)	Method	Limitations	Advantages
**Reflectance Photoplethysmography (rPPG) ** [36–39]	SC	Optical measurement of the back reflected transmitted light incident on a photodiode to detect variation in blood volume	• Sensor displacement • Motion artefacts • Studies only on stable newborns not in need of resuscitation	• Forehead perfusion better than peripheries • Easier placement of sensors • Less interference with resuscitation
**Dry ECG electrodes NeoBeat** [26–29]	SC	Light skin contact potential sensing	• Influence of environmental factors was not determined • Most studies on stable term babies not in resuscitation condition • Small sample size	• Quick to be placed and display the HR measurement • Easy to use • No need of skin preparation • Placement on upper abdomen should not interfere with chest compressions • Reported high correlation with reference conventional ECG monitor • Not influenced by motion • Suitable device for HR monitoring in both high- and low resource settings
**Handheld Doppler ultrasound (HhDU) ** [40–42]	SC	Detection of blood flow velocity through reflectance measurement of ultrasound	• Additional staff required to perform reading • Interference with resuscitation efforts • Not tested on preterm babies	• Whole team can hear heart rate • ECG does not always imply cardiac output whereas audible Doppler sounds do. • Quick takes 3 s and more accurate than auscultation and palpation HR • Equipment readily available in childbirth facilities in primary care • No financial implications
**Digital stethoscope (DS) ** [43–45]	SC	Digital recording & analysis of the acoustic waves of the heart	• Loss of sensor contact with precordium • Environmental noise	• Accuracy of DS HR is greater than chest auscultation and umbilical cord palpation
**Video Photoplethysmography (VPG) ** [34, 46–48]	NC	Video recording of skin to detect blood volume changes	• Motion artefact • Poor illumination • Not tested in low light environment	• Not affected by high frequency oscillation ventilation, gentle rocking movement • Non-obtrusive no discomfort, stress, pain, or epidermal stripping • No interference with X-rays • No impact on parent-child bonding
**Capacitive sensors ** [35, 49, 50]	NC	Non-contact electric potential sensing of the ECG	• Susceptible to power line interference • Movement artefacts	• Reduce the likelihood of irritation, allergy, or discomfort • Increased comfort • It has promise for introduction to bedding
**Piezoceramic sensors ** [51]	NC	Measurement of heart vibration through the piezoceramic effect	• Limited study to a single subject • Susceptibility to movement artefacts	• Non-contact so reduces the risk of skin disorders • No restriction of babies’ movement • Decreased mechanical and painful stimuli
**Laser Doppler vibrometer ** [48, 52]	NC	Optical based technique used to record the doppler frequency shift of a laser beam scattered from the chest wall through an interferometric based measurement	• Size • Complexity • Cost of equipment	• No contact • Continuous monitoring • Can be used in MRI • Reduced biohazards and risk of contamination • Reports simultaneously HR and RR • Can be used for prolonged monitoring

### Novel sensors using skin contact

There are other novel sensors identified that use skin contact, either continuous or intermittent, but are not attached to the babies such as: reflectance PPG, dry ECG electrodes, handheld Doppler device and digital stethoscope [8].

#### Reflectance Photoplethysmography(rPPG)

Reflectance photoplethysmography (rPPG) sensors have a photodiode (PD) that detects the diffusely reflected light after interacting with the tissues. A critical component detected is the cardiac synchronous variation in blood volume. The reflected light is amplified, filtered, and then sampled by an analogue-to-digital converter (ADC) at 2300 Hz and subsequently demodulated using a quadrature demodulator to recover the PPG signal [36].

In the three studies included in our review using rPPG the preferred area to place a PPG sensor was the forehead [36–38]. This is the most suitable measurement site and should not interfere with any of the care or resuscitation procedures [37]. There were other advantages of this choice of placement. Skin perfusion was better maintained in comparison with the peripheries, such as hands and feet, it was easy to access and did not increase the risk of hypothermia especially for extreme preterm babies. On the other hand, these babies tend to be delivered and placed into polythene bags for better temperature control. This makes it more difficult to access their hands and feet to place a monitoring sensor [36].

In the rPPG reported by Grubb et al., they used green light (525 nm) to optimize the amplitude of the pulsatile signal acquisition and modulated this at 575 Hz to suppress interference from ambient light [36]. The light source consisted of four 525 nm LEDs with two pairs on either side of a PD to provide even illumination of the tissue beneath the sensor. The detection device is based on three main blocks used to process the signals. The first included amplification and filtering. The second part involved using an analogue-to-digital converter (ADC) and a data –logging system which saved the data locally. The third part involved lock-in detection.

To determine the reliability of rPPG the authors calculated the positive percentage agreement (PPA) with HR by ECG and obtained values of 97.7% in the > 32 weeks gestational age (GA) group and 94.8% in < 32 weeks GA.

The reliability was negatively influenced by motion artefacts causing sensor displacement [36, 37]. The authors suggested methods to overcome these limitations by improving sensor fixation, using a suitable low pass filter applied to the HR data and adjusting in the averaging windows [36]. Ward et al. [38] recognized the role of qualitative studies with staff to optimize the device and the possibility of incorporating the PPG sensor in a hat.

There is a more recent study that echoes the previous studies’ results placing rPPG as a valid alternative to support transitioning, stabilization, and further interventions such as delayed cord clamping in newborn babies [39]. Authors clearly identify the need for further research to assess its reliability and accuracy in those babies with low perfusion states and in need of resuscitation.

#### Dry ECG electrodes NeoBeat

Several recent studies described this new promising method to determine and monitor HR in newborn babies. All authors used NeoBeat Newborn Heart Rate Meter (Laerdal Medical, Stavanger, Norway, LGH-532-0033), a non-disposable dry-electrode ECG and compared it with conventional wet adhesive ECG electrodes and some of them with PO. The device uses dry stainless-steel electrodes embedded in a spring-elastic buckle which is placed over the newborns chest or abdomen to measure electrical conductance ECG [26–29].

NeoBeat has several advantages compared to conventional ECG monitoring. It is easy to place and quick to display a HR measurement [27, 28]. The lowest time from placement to display was 2.5 s versus 58.5 s for PO [28]. It does not need skin preparation and placement on the upper abdomen does not interfere with resuscitation manoeuvres such as chest compressions. NeoBeat, dry ECG electrode’s HR measurements corelate well with conventional ECG so it is considered reliable and accurate [26–29]. Due to its reduced cost, it can be used both in high and low resource settings.

Limitations of these studies were due the small sample sizes and selection of only stable babies. Influence of certain factors such as resuscitation efforts and poor perfusion was not determined. There was variability in the averaging times of NeoBeat dry ECG electrode and conventional ECG monitors [26].

These authors agree that future studies are needed to determine the reliability and accuracy of NeoBeat Dry ECG electrodes during deliveries requiring intensive resuscitation, but all recognise its potential to improve outcomes [26, 27, 29].

#### Handheld doppler

Handheld Doppler ultrasound (HhDU) to detect HR on babies at birth has been described in three studies included in our literature review [40–42]. Bidirectional handheld Doppler is usually used for measuring arterial and venous blood flow in the extremities and for detecting foetal or neonatal HR. Blood flow velocity is detected through the ultrasound which is transmitted from probe to patient body and is reflected by the blood.

The transmitter transducer converts amplified high frequency electrical oscillations into ultrasound which is transmitted to the patient. The ultrasound passes through biological tissue and is reflected by moving objects (i.e., the beating of the neonatal heart). The reflected ultrasound is converted back into electrical signals by the receiving transducer, and the resulting Doppler shifted signals are amplified and replayed as audible sound through a speaker.

Overall HR by HhDU correlated well with HR by ECG and is considered a quick solution to determine HR at birth. Other advantages included that the whole team could hear the heartbeat, it was faster than auscultation and palpation and the equipment was readily available at childbirth facilities so there were no financial implications.

The limitations of this method are that it requires enhanced training, an extra staff member to perform the task and it can interfere with resuscitation efforts [40–42].

#### Digital stethoscopes

Digital Stethoscopes have been used to record heart and respiratory sounds in infants and children. Some studies have used the recordings as part of investigation of techniques for analysis of heart murmurs [53, 54]. A few studies which have investigated using digital stethoscopes in the delivery room have noted some limitations when assessing heart rate in crying babies [43, 44].

Digital stethoscopes in paediatric medicine have potential to reduce the listeners subjectivity and can be used in telemedicine for increasing specialist access; computer-aided diagnostic programs/algorithms for clinical diagnosis based on auscultation, home-based monitoring of cardiorespiratory conditions and clinical teaching [45].

Further studies are necessary to investigate the reliability and accuracy of using digital stethoscopes in detecting newborn heart rate at birth [43].

### Non-contact novel sensor-based methods

Non-contact sensors used to detect HR in babies identified by the systematic review were: video photoplethysmography (VPG) [34, 46, 47, 55], capacitive electrodes [35, 49, 50], piezo-ceramic [51], piezo- electric sensors [56] and laser Doppler vibrometer [52].

#### Video Photoplethysmography (VPG)

Video photoplethysmography is a contactless optical measurement technique that can be used to detect blood volume change in the vascular bed of a certain skin area. These changes affect light transmission and reflectance. The cardiovascular pulse waves cause changes in the volume of arterioles which result in minute pulsatile skin colour changes. These ‘micro-blushes” are invisible to the human eye but can be detected by high-definition cameras [47].

Aarts et al. have used this technology in their pilot study published in 2013 [46]. The cameras were placed at 1 m distance from the babies and recorded for 1 to 5 min in the ambient light of NICU, aiming at uncovered body parts and recording 300 × 300 pixel 8-bit video at 30fps to be saved in uncompressed AVI format for later analysis.

Using a custom-built graphical user interface, the researcher manually selected a video frame and defined it as a first region of interest (ROI). Part of the signal processing utilized a template for global motion tracking of the infant (IMAQ 3 match pattern, National Instruments, Austin, TX). Within the first ROI a second, smaller ROIs containing skin was selected. They calculated the average pixel value (PV) of each frame in the ROIs. They obtained a PV(t)signal by plotting each frames’ PV of ROIs. The analysed channel was the green channel because it offered the strongest colour. The cardiovascular pulse wave traveling through the cutaneous blood vessels caused the variations in PV(t) of the ROIs as a function of time are caused by minute colour changes in the skin. They used the red channel, with the lowest reflectance variation to correct the green channel signal for illumination intensity changes.

The systematic review identified three other studies using similar experiments. All were small studies on both term and preterm infants in NICU that evaluated the accuracy of camera photoplethysmography to detect and monitor HR in neonates [34, 46, 47, 55]. All studies reported a high correlation between the VPG HR and ECG or PO HR with limits of agreement as high as 95% [46]. Differences were reported close to medical standards with values from +/- 4 beats per minute (bpm) to +/- 5.48 bpm.

The advantages of VPG were improved comfort especially if prolonged periods of observation are required [47], it was pain free and it did not interfere with other equipment (e.g. X-Ray machines). Furthermore, it can be used on babies with dark skin (a known limitation with photoplethysmography) and during gentle movement caused by high frequency oscillation ventilation and rocking movements (in mum’s arms) [57]. Most importantly, it was considered not to have a negative impact on parent-child bonding [46]. VPG has the potential for remote healthcare monitoring and could be used in low resource settings due to reduced costs and is an easy to deploy system [47, 55].

Limitations and obstacles in use of VPG were due to loss of recording due to motion artifacts [46–48], poor ambient illumination (all 4 studies) and regular interaction between clinical staff and the baby [34].

Authors explored several strategies to overcome these limitations using improved extraction algorithms, higher definition cameras [46], better light conditions [46, 47] and developing systems to reduce movement artifact [55].

#### Capacitive sensors

Capacitive integrated sensing measures electro-physiological signals without needing direct skin contact. Sensors can be imprinted on materials that can be worn by a person, or embedded in objects surrounding the body, such as a mattress or a chair. This method has shown enormous potential in many applications to acquire an ECG signal. To reduce the incomplete coverage caused by motion and positioning researchers propose an array of sensors embedded in a mattress [49, 58]. The advantage of these sensors is that they reduce the likelihood of skin irritation, allergy, and discomfort due to painful stimuli. Unfortunately, several studies have concluded that due to suboptimal sensor coupling and movement artefacts they had high positive predictive value but with low sensitivity in obtaining a HR measurement. The same studies mention strategies to overcome these limitations by achieving a better design of the sensor matrix and using a smaller number of layers when embedded [49, 50, 58].

A recent paper by Dore et al. [35] reports the design and development of a technological solution to provide real time ECG data for clinicians within the delivery room using non-contact electric potential sensors (EPS) embedded in a neonatal intensive care unit mattress. The electric potential sensor (EPS) is an electrometer-based amplifier insulating electrode that does not require galvanic contact with the body to acquire biopotential signals. It operates with displacement currents and the traditional electrode-skin interface is replaced with a dielectric material. The proof-of-concept tests were carried out both in vivo and in vitro using simulated and human cardiac signals. Two materials were considered for embedding the sensors within a NICU mattress in this study: a silver conductive polymer ink (Fabink-TC-C4001) and a self-adhesive conductive textile fabric (MOS TitanRF). The EPS sensor was designed with an external bias circuitry that does not compromise the input impedance of the sensors. The feedback loops use guarding, bootstrapping and neutralization to enhance the input impedance, reduce its capacitance and support the sensor’s stability. This prototype system including the EPS sensor and textile-based electrodes has successfully recorded a high-quality ECG both from the simulated and human source. The quality assessment of the signal confirmed that high quality ECG recording was achieved in the presence of interference layers and an air gap, with reliable and repeatable ECG waveforms. Authors concluded that this device has high potential to automatically acquire the HR of a newborn within 10 s of delivery without needing the attachment of additional sensors. This is considered a safe, rapid, and reliable technological solution with great potential to improve the newborn mortality and morbidity outcomes.

#### Piezoceramic sensor

The piezoceramic sensor is described by Nukaya et al. [51, 59]. Four sensors are attached between the legs of the neonatal bed, employing a resin plate to increase the bending of the support surface and therefore the sensitivity, to account for the low weight of the neonate. Micro vibrations from the high frequency component of the heartbeat were then identified, to avoid the problem of environmental noise in the fundamental frequency region (2–3 Hz) of the neonatal heartbeat. Other bio signals recognized were respiration and body movements. To determine the validity of the system, electrocardiogram and respiratory waveform output from a conventional bedside monitor were used as references.

The heartbeat and respiration signals detected by the piezoceramic sensor were similar to those detected by the monitor. Correlation coefficient of HR was high in between the values showed by the piezoceramic sensor and those of the conventional monitor R = 0.91 [51]. The limitations of their study were the experimental design and small number of subjects as it included only one preterm baby. The technology has potential for long term monitoring offering all the advantages of non-contact sensors such as reducing the risk of skin disorders and decreased mechanical and painful stimulation [51].

#### Laser doppler vibrometer

The laser Doppler vibrometer is an optical measurement method for continuous monitoring of both HR and RR. The non-contact method uses a class II B laser (wavelength 632.8 nm, power < 1 mW) pointing towards a thoracic site near the nipple area to measure the chest wall movements, associated with inspiratory/expiratory activities of the lungs and with the mechanical pumping action of the heart. It has all the advantages of the other non-contact devices, reducing the risk of infection, by not affecting the skin integrity, and reducing the discomfort and the painful stimuli associated with adhesive sensors. Several experimental studies looked at correlation to HR by ECG with differences in between the two methods of less than 6% [52] but also to RR monitoring compared to spirometer data which was less than 3% [48, 60]. Authors considered that the limitations were due to large size, complexity, and associated costs [52].

### Respiratory rate (RR) monitoring using pulse oximetry

RR is a key vital sign in young children and increased respiratory rate (tachypnoea) is an important sign suggesting several acute conditions such [10, 61, 62] as infections but also a wide range of congenital respiratory, cardiac and neurological disorders [63]. Monitoring RR is especially important to identify sick and vulnerable infants and children offering early diagnosis and treatment of the disease. At present continuous monitoring of RR is by measurement of exhaled breath and thoracic effort [63].

There are a variety of sensors used to monitor exhaled breath. RR can be derived from the oronasal moisture sensors which measure the fluctuation of humidity with respiration. Thermistor sensors determine a derived RR by measuring the fluctuation of temperature with respiration in the upper airway. Barometric pressure sensors, pressure transductors or airflow velocity sensors measure the air pressure in the oronasal cavity. The RR is derived from the fluctuations in air pressure with respiration. Also using exhaled breath, RR can be obtained using capnography sensors which measure the fluctuations in carbon dioxide with respiration [63].

RR can also be derived by measuring thoracic expansion. Thoracic impedance sensors, widely used in intensive care, measure the variation in thoracic impedance with respiration using weak electronic signals emitted from the ECG leads. Respiratory inductance plethysmography uses the changes in inductance in chest and abdominal bands caused by the changes in chest and abdominal circumference during breathing and is used in sleep laboratories.

These methods are invasive and difficult for regular clinical care outside the intensive care settings. Therefore, there is a high interest in developing a non-invasive technological solution for continuous RR monitoring [16, 19].

Pulse oximetry is already widely used for monitoring of the oxygen saturations (SpO2) in all clinical settings and even at home. Generally, the placement of the pulse oximeter sensor is well tolerated by infants and children.

Pulse oximeters produce a plethysmogram (pleth) trace reflecting the instantaneous volume of blood in the extremity under the probe. Changes in the respiratory effort can affect the pleth trace due to multiple mechanisms involved in cardiorespiratory interactions [21]. Using a low pass filtering technique (LPF), the dominant heart rate component of the pleth trace can be removed to yield a trace that contains the lower frequency components [64, 65]. The filtered pleth trace can thus be used to derive the respiratory rate (RR) [16, 17, 19]. Processing of the pleth has been performed using custom made software developed with MATLAB (The MathWorks Inc., Natick, MA, USA) [16–19]. The LPF cut-off frequency used was at half the pulse rate calculated in sequential epochs of length 1 to 2 min duration; the filter characteristics give a steep roll-off to remove the pulse rate component.

Several studies have demonstrated that RR can be extracted accurately from the pleth trace of standard pulse oximeters [16, 18, 19]. Their findings have great implications for clinical practice offering a solution for non-invasive continuous RR monitoring in different settings [19]. The technique has potential to be used for home monitoring patients with chronic respiratory disease, as well as identifying early warning signs for respiratory infection in patients with cystic fibrosis. Early diagnosis may result in prompter care provision, quicker recovery, and better patient outcomes [66].

## Discussions and conclusions

This overview highlists an overall current trend of aiming to minimize patient contact and allow non-obtrusive measurements of vital signs especially in highly vulnerable populations such as newborn and preterm babies. Researchers with different areas of expertise are collaborating to develop non-invasive or minimally invasive sensors which are accurate, reliable and rapid [8, 9, 67].

Non-contact ECG sensors have several advantages and can guide not only resuscitation but also other perinatal interventions, such as delayed cord clamping [8]. Future studies, using novel technologies, may clarify the role of placental transfusion in cardiovascular adaptation after birth [8, 39, 68].

RR is another key vital sign used as a valuable marker of illness in vulnerable infants [10, 11]. Current methods to continuously monitor RR are problematic for regular clinical use as they can be invasive and limited to intensive care units. The novel approach presented uses pulse oximetry and low pass filters to extract RR from the pleth trace. Multiple studies have proven that this is feasible method with clear potential for aiding clinical practice; for example, the approach has potential to be used in the home for monitoring neonates and children with chronic respiratory disease, as well as in overnight studies in children with suspected obstructive sleep apnoea and monitoring for early warning of respiratory infection in children with cystic fibrosis [15, 19, 69].

Recent studies show promising results for heart rate monitoring using sensors such as dry ECG electrodes [26–29], both video [70] and reflectance photoplethysmography [39], capacitive sensors [35, 58] handheld ultrasound [71] and laser vibrometer [72]. Other technologies such as piezoceramic sensors [59] and digital stethoscopes [43, 44, 73] have been less investigated.

Problems potentially limiting the use of the novel sensors can be related to movement artefacts and bad sensor coupling which may result in poor-quality recordings and intermittent measurements [8, 9, 67]. Strategies to overcome these limitations have been discussed by several authors. These include embedding the sensors in a support system situated close to the skin, reducing the numbers of layers, and developing a better design for the sensor matrix [49]. There is also keen interest in developing advanced signal-processing algorithms [8, 9].

Novel sensors should be accurate, reliable, durable, easy to use and cost-effective; furthermore, studies would be needed to investigate whether new sensors, when compared with current established methods, show improved performance. Developing new sensors is likely to require a multidisciplinary research team including for example engineers, software developers, physicists together with clinical team involved in the care of the newborn baby, midwives, obstetricians, paediatricians, neonatal doctors and nurses [8, 67, 68]. For novel sensors to be accepted for clinical use studies would need to examine their performance under clinical conditions and in different clinical areas (NICU, delivery suits, community birthing centres).

HR and RR are key vital signs used in resuscitation and monitoring of newborn babies [7–9]. Developing novel monitoring technology has the potential to improve clinical practice and result in better short- and long- term neonatal health outcomes [8, 9, 67].

## Data Availability

Data regarding the reviewed publications is available to review.

## Abbreviations

HR: Heart rate
RR: Respiratory rate
PO: Pulse oximetry
ECG: Electrocardiography
PPG: Photoplethysmography
RIP: Respiratory inductive plethysmography
NICU: Neonatal intensive care unit
HDU: High dependency unit
LPF: Low pass filtering
rPPG: Reflectance photoplethysmography
SC: Skin contact
NC: Non-contact
HhDU: Hand held doppler ultrasound
DS: Digital stethoscope
VPG: Video plethysmography
ROI: Region of interest
PV: Pixel value
EPS: Electric potential sensor
SoO2: Oxygen saturations
SQI: Signal quality index

## References

[1] Organisation WH. Newborns:improving survival and well-being. Vol. 2021. 2020. Available from: https://www.who.int/news-room/fact-sheets/detail/newborns-reducing-mortality.

[2] RCPCH. Infant mortality RCPCH State of Child Health. 2020. Available from: https://stateofchildhealth.rcpch.ac.uk/evidence/mortality/infant-mortality/.

[3] Wyllie J, Perlman JM, Kattwinkel J, Wyckoff MH, Aziz K, Guinsburg R. Neonatal Resuscitation Chapter Collaborators. Part 7: Neonatal resuscitation: 2015 International Consensus on Cardiopulmonary Resuscitation and Emergency Cardiovascular Care Science with Treatment Recommendations. Resuscitation. 2015;95:e169–201. 10.1016/j.resuscitation.2015.07.045. 10.1016/j.resuscitation.2015.07.045 26477424

[4] Wyckoff MH, Wyllie J, Aziz K, de Almeida MF, Fabres J, Fawke J (2020). Neonatal Life Support: 2020 International Consensus on Cardiopulmonary Resuscitation and Emergency Cardiovascular Care Science With Treatment Recommendations. Circulation.

[5] Wyllie J, Perlman JF, Kattwinkel J, Atkins DL, Chameides L, Goldsmith JP (2010). Part 11: Neonatal resuscitation: 2010 International consensus on cardiopulmonary resuscitation and emergency cardiovascular care science with treatment recommendations. Resuscitation..

[6] Perlman JM, Wyllie J, Kattwinkel J, Wyckoff MH, Aziz K, Guinsburg R (2015). Part 7: Neonatal Resuscitation: 2015 International Consensus on Cardiopulmonary Resuscitation and Emergency Cardiovascular Care Science With Treatment Recommendations (Reprint). Pediatrics..

[7] Phillipos E, Solevag AL, Pichler G, Aziz K, van Os S, O’Reilly M (2016). Heart Rate Assessment Immediately after Birth. Neonatology..

[8] Anton O, Fernandez R, Rendon-Morales E, Aviles-Espinosa R, Jordan H, Rabe H (2019). Heart Rate Monitoring in Newborn Babies: A Systematic Review.

[9] Kevat AC, Bullen DVR, Davis PG, Kamlin COF (2017). A systematic review of novel technology for monitoring infant and newborn heart rate. Acta Paediatr.

[10] Fleming S, Thompson M, Stevens R, Heneghan C, Plüddemann A, Maconochie I (2011). Normal ranges of heart rate and respiratory rate in children from birth to 18 years of age: a systematic review of observational studies. Lancet..

[11] Simoes EA, Roark R, Berman S, Esler LL, Murphy J (1991). Respiratory rate: measurement of variability over time and accuracy at different counting periods. Arch Dis Child.

[12] Edwards MO, Kotecha SJ, Kotecha S (2013). Respiratory distress of the term newborn infant. Paediatr Respir Rev.

[13] Benning A, Ghaleb M, Suokas A, Dixon-Woods M, Dawson J, Barber N (2011). Large scale organisational intervention to improve patient safety in four UK hospitals: mixed method evaluation. BMJ..

[14] AL-Khalidi FQ, Saatchi R, Burke D, Elphick H, Tan S (2011). Respiration rate monitoring methods: A review. Pediatr Pulmonol..

[15] Wertheim D, Olden C, Symes L, Rabe H, Seddon P (2013). Monitoring respiration in wheezy preschool children by pulse oximetry plethysmogram analysis. Med Biol Eng Comput.

[16] Wertheim D, Olden C, Symes L, Rabe H, Seddon P (2013). Monitoring respiration in wheezy preschool children by pulse oximetry plethysmogram analysis. Med Biol Eng Comput..

[17] Seddon P, Kouman SS, Wertheim D. Home respiratory monitoring in infants born preterm using pulse oximeter plethysmogram analysis. Eur Respir J. 2015;46. Available from: 10.1183/13993003.congress-2015.PA1259.

[18] Seddon P, Olden C, Wertheim D, Banks S, Kapur A. Monitoring respiratory rate from pulse oximetry during treatment of acute severe wheeze in young children. European Resp J. 2017;50. Available from: 10.1183/1393003.congress-2017.PA1329.

[19] Seddon P, Sobowiec-Kouman S, Wertheim D (2018). Infant home respiratory monitoring using pulse oximetry. Arch Dis Child.

[20] Schmiech D, Muller S, Diewald AR. 4-Channel I/Q-Radar System For Vital Sign Monitoring In A Baby Incubator. 2018 19th International Radar Symposium (IRS). 2019, pp. 1–9. 10.23919/IRS.2018.8448163.

[21] Gleichauf J, Herrmann S, Hennemann L, Krauss H, Nitschke J, Renner P (2021). Automated Non-Contact Respiratory Rate Monitoring of Neonates Based on Synchronous Evaluation of a 3D Time-of-Flight Camera and a Microwave Interferometric Radar Sensor. Sensors..

[22] Khan F, Ghaffar A, Khan N, Cho SH (2020). An Overview of Signal Processing Techniques for Remote Health Monitoring Using Impulse Radio UWB Transceiver. Sensors..

[23] Kim JD, Lee WH, Lee Y, Lee HJ, Cha T, Kim SH (2019). Non-contact respiration monitoring using impulse radio ultrawideband radar in neonates. R Soc Open Sci..

[24] Mahbub I, Shamsir S, Pullano SA, Islam SK (2019). Low-power low-data-rate IR-UWB transmitter for paediatric apnoea monitoring system. IET Circuits Devices Syst..

[25] Huang X, Sun L, Tian T, Huang Z, Clancy E. Real-time non-contact infant respiratory monitoring using UWB radar. 2015 IEEE 16th International Conference on Communication Technology (ICCT), 2015, pp. 493–6. 10.1109/ICCT.2015.7399885.

[26] Pike H, Eilevstjønn J, Bjorland P, Linde J, Ersdal H, Rettedal S (2021). Heart rate detection properties of dry-electrode ECG compared to conventional 3-lead gel-electrode ECG in newborns. BMC Res Notes..

[27] Bush JB, Cooley V, Perlman J, Chang C (2021). NeoBeat offers rapid newborn heart rate assessment. Arch Dis Child Fetal Neonatal Ed..

[28] van Twist E, Salverda HH, Te Pas AB. Comparing pulse rate measurement in newborns using conventional and dry-electrode ECG monitors. LID – 10.1111/apa.16242. 10.1111/apa.16242 PMC9303717 34981852

[29] Rettedal S, Eilevstjønn J, Kibsgaard A, Kvaløy JT, Ersdal H (2021). Comparison of Heart Rate Feedback from Dry-Electrode ECG, 3-Lead ECG, and Pulse Oximetry during Newborn Resuscitation. Children (Basel)..

[30] Mizumoto H, Tomotaki S, Shibata H, Ueda K, Akashi R, Uchio H (2012). Electrocardiogram shows reliable heart rates much earlier than pulse oximetry during neonatal resuscitation. Pediatr Int.

[31] O’Donnell CP, Kamlin CO, Davis PG, Morley CJ (2005). Feasibility of and delay in obtaining pulse oximetry during neonatal resuscitation. J Pediatr.

[32] Katheria AC, Brown MK, Rich W, Arnell K (2017). Providing a Placental Transfusion in Newborns Who Need Resuscitation. Front Pediatr.

[33] Iglesias B, Rodriguez MJ, Aleo E, Criado E, Herranz G, Moro M (2016). [Pulse oximetry versus electrocardiogram for heart rate assessment during resuscitation of the preterm infant]. Pulsioximetria frente al monitor de electrocardiograma para la determinacion de la frecuencia cardiaca durante la reanimacion del recien nacido pretermino. An Pediatr (Barc).

[34] Villarroel M, Guazzi A, Jorge J, Davis S, Watkinson P, Green G (2014). Continuous non-contact vital sign monitoring in neonatal intensive care unit. Healthc Technol Lett.

[35] Dore H, Aviles-Espinosa R, Luo Z, Anton O, Rabe H, Rendon-Morales E (2021). Characterisation of Textile Embedded Electrodes for Use in a Neonatal Smart Mattress Electrocardiography System. Sensors (Basel)..

[36] Grubb MR, Carpenter J, Crowe JA, Teoh J, Marlow N, Ward C (2014). Forehead reflectance photoplethysmography to monitor heart rate: preliminary results from neonatal patients. Physiol Meas.

[37] Mann C, Ward C, Grubb M, Tech J, Crowe J, Hayes Gill B (2011). Can we improve delivery room monitoring for newborns? A novel photoplethysmographic heart rate monitor evaluated among stable NICU infants. Arch Dis Child Fetal Neonatal Ed..

[38] Ward C, Teoh J, Crowe J, Sharkey D, Grubb M, Marlow N (2010). A green light for improved resuscitation. Midwives..

[39] Henry C, Shipley L, Ward C, Mirahmadi S, Liu C, Morgan S (2021). Accurate neonatal heart rate monitoring using a new wireless, cap mounted device. Acta Paediatr.

[40] Dyson A, Jeffrey M, Kluckow M (2017). Measurement of neonatal heart rate using handheld Doppler ultrasound. Arch Dis Child Fetal Neonatal Ed..

[41] Goenka S. Precordial Doppler ultrasound achieves earlier and more accurate newborn heart rates in the delivery room. In2013 AAP National Conference and Exhibition 2013 Oct 25. Am Acad Paediatr. p. E-PAS2014:3843.590.

[42] Shimabukuro R, Takase K, Ohde S, Kusakawa I (2017). Handheld fetal Doppler device for assessing heart rate in neonatal resuscitation. Pediatr Int.

[43] Gaertner VD, Kevat AC, Davis PG, Kamlin COF (2017). Evaluation of a digital stethoscope in transitioning term infants after birth. Arch Dis Child Fetal Neonatal Ed.

[44] Treston BP, Semberova J, Kernan R, Crothers E, Branagan A (2019). Assessment of neonatal heart rate immediately after birth using digital stethoscope, handheld ultrasound, and electrocardiography: an observational cohort study. Arch Dis Child Fetal Neonatal Ed..

[45] Ramanathan A, Zhou L, Marzbanrad F, Roseby R, Tan K, Kevat A (2019). Digital stethoscopes in paediatric medicine. Acta Paediatr.

[46] Aarts LAM, Jeanne V, Cleary JP, Lieber C, Nelson JS, Bambang Oetomo S (2013). Non-contact heart rate monitoring utilizing camera photoplethysmography in the neonatal intensive care unit - A pilot study. Early Human Dev.

[47] Mestha LK, Kyal S, Xu B, Lewis LE, Kumar V, Towards continuousmonitoring of pulse rate in neonatal intensive care unit with a webcam. Vol. (2014). Conference proceedings : Annual International Conference of the IEEEEngineering in Medicine and Biology Society.. Annu Int Conf IEEE Eng Med Biol Soc.

[48] Scalise L, Marchionni P, Ercoli I, Tomasini EP (2012). Laser measurement of respiration activity in preterm infants: Monitoring of peculiar events. AIP Conference Proc.

[49] Atallah L, Serteyn A, Meftah M, Schellekens M, Vullings R, Bergmans JW (2014). Unobtrusive ECG monitoring in the NICU using a capacitive sensing array. Physiol Meas.

[50] Kato T, Ueno A, Kataoka S, Hoshino H, Ishiyama Y (2006). An application of capacitive electrode for detecting electrocardiogram of neonates and infants. Conf Proc IEEE Eng Med Biol Soc.

[51] Nukaya S, Sugie M, Kurihara Y, Hiroyasu T, Watanabe K, Tanaka H (2014). A noninvasive heartbeat, respiration, and body movement monitoring system for neonates. Artificial Life Robotics.

[52] Marchionni P, Scalise L, Ercoli I, Tomasini EP. An optical measurement method for the simultaneous assessment of respiration and heart rates in preterm infants. The Review of scientific instruments. 2013;84. Available from: http://ovidsp.ovid.com/ovidweb.cgi?T=JS&PAGE=reference&D=med7&NEWS=N&AN=24387410. 10.1063/1.4845635 24387410

[53] Lai LS, Redington AN, Reinisch AJ, Unterberger MJ, Schriefl AJ (2016). Computerized Automatic Diagnosis of Innocent and Pathologic Murmurs in Pediatrics: A Pilot Study. Congenit Heart Dis.

[54] Takahashi K, Ono K, Arai H, Adachi H, Ito M, Kato A (2021). Detection of Pathologic Heart Murmurs Using a Piezoelectric Sensor. Sensors (Basel)..

[55] Scalise L, Bernacchia N, Ercoli I, Marchionni P (2012). Heart rate measurement in neonatal patients using a webcamera. IEEE International Symposium on Medical Measurements and Applications Proceedings.

[56] Sato S, Ishida-Nakajima W, Ishida A, Kawamura M, Miura S, Ono K (2010). Assessment of a new piezoelectric transducer sensor for noninvasive cardiorespiratory monitoring of newborn infants in the NICU. Neonatology..

[57] Fine J, Branan KL, Rodriguez AJ, Boonya-ananta T, Ajmal, Ramella-Roman JC (2021). Sources of Inaccuracy in Photoplethysmography for Continuous Cardiovascular Monitoring. Biosensors..

[58] Aviles-Espinosa R, Rendon-Morales E, Luo Z, Dore H, Anton O, Rabe H (2019). Neo-SENSE: a non-invasive smart sensing mattress for cardiac monitoring of babies. IEEE Sensors Applications Symposium (SAS).

[59] Nukaya S, Shino T, Kurihara Y, Watanabe K, Tanaka H (2012). Noninvasive Bed Sensing of Human Biosignals Via Piezoceramic Devices Sandwiched Between the Floor and Bed. IEEE Sens J.

[60] Scalise L (2011). Lasermonitoring of respiration activity in preterm infants: Monitoring of peculiar events. Lasers Med Sci.

[61] Yapicioglu H, Ozlu F, Sertdemir Y (2015). Are vital signs indicative for bacteremia in newborns?. J Matern Fetal Neonatal Med.

[62] Mortensen N, Augustsson JH, Ulriksen J, Hinna UT, Schmöizer GM, Solevagå AL (2017). Early warning- and track and trigger systems for newborn infants: A review. J Child Health Care.

[63] Ginsburg AS, Lenahan JL, Izadnegahdar R, Ansermino JM (2018). A Systematic Review of Tools to Measure Respiratory Rate in Order to Identify Childhood Pneumonia. Am J Respir Crit Care Med.

[64] Leonard P, Beattie TF, Addison PS, Watson JN (2003). Standard pulse oximeters can be used to monitor respiratory rate. Emerg Med J.

[65] Foo JY, Wilson SJ (2005). Estimation of breathing interval from the photoplethysmographic signals in children. Physiol Meas.

[66] Trubey R, Huang C, Lugg-Widger FV, Hood K, Allen D, Edwards D (2019). Validity and effectiveness of paediatric early warning systems and track and trigger tools for identifying and reducing clinical deterioration in hospitalised children: a systematic review. BMJ Open..

[67] Johnson PA, Schmölzer GM (2020). Heart Rate Assessment during Neonatal Resuscitation. Healthc Basel Switz..

[68] Anton O, Fernandez R, Rendon-Morales E, Aviles-Espinosa R, Jones CJ, Rabe H (2020). Functionality and acceptability of a novel non-invasive neonatal heart rate monitoring device: a qualitative study of healthcare professionals. BMJ Innovations.

[69] Wertheim D, Olden C, Savage E, Seddon P (2009). Extracting respiratory data from pulse oximeter plethysmogram traces in newborn infants. Arch Dis Child.

[70] Selvaraju V, Spicher N, Wang J, Ganapathy N, Warnecke JM, Leonhardt S (2022). Continuous Monitoring of Vital Signs Using Cameras: A Systematic Review. Sensors..

[71] Manzar S, Bhat R (2022). Feasibility of handheld ultrasound to assess heart rate in newborn nursery. Resuscitation..

[72] Antognoli L, Moccia S, Migliorelli L, Casaccia S, Scalise L, Frontoni E (2020). Heartbeat Detection by Laser Doppler Vibrometry and Machine Learning. Sensors..

[73] Kevat AC, Dawson J, Davis PG, Kamlin CO (2015). Evaluation of a digital stethoscope and smart device technology for assessment of heart rate in the newborn infant. Arch Dis Child Fetal Neonatal Ed.

